# Bibliometric Analysis of Small-Cell Lung Cancer in Geriatric Populations

**DOI:** 10.7759/cureus.82688

**Published:** 2025-04-21

**Authors:** Dyuthi Nallavolu, Sydney N Vaughn, Latha Ganti

**Affiliations:** 1 Research, Orlando Science School, Orlando, USA; 2 Public Health, Brown University, Providence, USA; 3 Medical Sciences, The Warren Alpert Medical School of Brown University, Providence, USA; 4 Research, Orlando College of Osteopathic Medicine, Winter Garden, USA; 5 Emergency Medicine and Neurology, University of Central Florida, Orlando, USA

**Keywords:** bibliometric analysis, chemotherapy, oncology, pulmonology research, small cell lung cancer (sclc)

## Abstract

This study aimed to analyze the literature published about small-cell lung cancer (SCLC) in geriatric populations on a global scale from 1979 to 2023. This bibliometric analysis was performed by gathering data from the Web of Science Core Data Collection, using keywords such as small-cell lung cancer, SCLC, extensive-stage SCLC (ES-SCLC), and oat cell cancer. The search was further refined to target an older adult population by incorporating keywords such as 65+ years of age, geriatric patients, and baby boomers. These articles were then analyzed using VOSviewer. Maps were generated using sorting methods, focusing specifically on organizations, countries, and keywords. More than 7,710 articles spanning from 1979 to 2023 were analyzed. The United States, the People’s Republic of China, and Japan produced the most publications on small-cell lung cancer in geriatric patients. When analyzing keywords used in studies on small-cell lung cancer in geriatric patients, many were related to the treatment and therapy of the disease. This bibliometric analysis shows that as time progresses, the focus of the United States, the People’s Republic of China, and Japan is to increase research on therapeutics and treatments to help remedy small-cell lung cancer in order to decrease fatal lung cancer diagnoses in geriatric patients. If these countries continue their research efforts as before, more advancements in treatment can be made to ultimately find a cure for small-cell lung cancer, particularly impacting geriatric populations.

## Introduction and background

As of 2020, there were approximately 2.2 million new cases of lung cancer in the world. In the United States specifically, there are 234,580 lung cancer diagnoses. Approximately 116,310 of these cases are in men, while 118,270 are in women. As a result, the risk of being diagnosed with lung cancer in the United States is about one in 16-17 cases. Cigarette smoking is one of the main contributing factors that can lead to an early onset of lung cancer [1]. As stated by the CDC, smoking is linked to about 80-90% of lung cancer deaths in the United States [2].

One specific subtype of lung cancer is small-cell lung cancer, which impacts around 250,000 people globally per year. Unlike non-small-cell lung cancer, which slowly progresses, small-cell lung cancer is far more aggressive and is characterized by the rapid development of cells that quickly spread throughout the body [3]. According to the National Cancer Institute, small-cell lung cancer is less common than non-small-cell cancer, accounting for only 15% of all lung cancer cases. Lung cancer primarily affects the geriatric population, with onset times of around 70 years of age [4]. There is a great disparity between decreased cancer aggressiveness in an individual patient and a high rate of cancer mortality in older age groups [5]. This disparity may be due to the survival data being confounded by special problems common to geriatric populations, e.g., comorbidity, “poly-pharmacy” (i.e., the common practice of prescribing numerous drugs in older people), physician or family bias regarding diagnosis and treatment in the elderly, and age-associated life stresses that may be as basic as the inability to get to a medical center for treatment [5]. This means that this specific population usually has poor diagnoses and a higher possibility of recurrence of the cancer, even with treatment [6]. Despite the high chance that new malignancies will occur in the elderly who have cancer, cancer in the elderly is often poorly treated, and the behavior of cancer is highly misunderstood [7]. However, if it is identified in the early stages, patients have a 60-70% chance of improving their health [8]. Bibliometric analysis is a quantitative and effective approach used to assess publication patterns and research trends within a specific field or topic of interest. This study utilizes a bibliometric approach to analyze these trends related to small-cell lung cancer in older adult populations.

## Review

Methods

Bibliometric analysis is a method used to analyze large datasets to identify trends and gain information through articles and journals. Bibliometric analysis is used to uncover historical or emerging trends, measure the impact of specific studies or authors, or identify influential journals or institutions [9]. Bibliometric methods for the medical literature were followed [10].

The data analyzed in this study were obtained from the Web of Science Core Collection, limited to articles and review papers. The database was searched from its inception to March 1, 2025, with no language restrictions applied. The analysis focused on the countries where the research was conducted, the contributing authors, and their affiliated institutions. All selected publications were evaluated using VOSviewer (Leiden, Netherlands: Leiden University), applying the association strength method for analysis.

The words submitted into the advanced search feature included the following: TS=((Small cell lung cancer OR small-cell lung cancer OR SCLC OR ES-SCLC OR extensive stage small cell lung cancer OR extensive-stage small-cell lung cancer OR extensive stage small cell lung cancer OR oat cell cancer OR neuroendocrine tumours OR neuroendocrine tumors OR small cell tumor OR small cell tumours OR small cell carcinoma OR combined small cell carcinoma OR neuroendocrine cell cancer OR oat cell type lung cancer) AND (elderly OR elders OR 65 and older OR baby boomers OR seniors OR senior citizen OR geriatric patients OR geriatric OR 65+)) and a period of 1979-2023. TS=((Small cell lung cancer OR small-cell lung cancer OR SCLC OR ES-SCLC OR extensive stage small cell lung cancer OR extensive-stage small-cell lung cancer OR extensive stage small cell-lung cancer OR oat cell cancer OR neuroendocrine tumours OR neuroendocrine tumors OR small cell tumor OR small cell tumours OR small cell carcinoma OR combined small cell carcinoma OR neuroendocrine cell cancer OR oat cell type lung cancer) AND (elderly OR elders OR 65 and older OR baby boomers OR seniors OR senior citizen OR geriatric patients OR geriatric OR 65+)) AND PY=(1979-2023)).

Results

**Figure 1:**
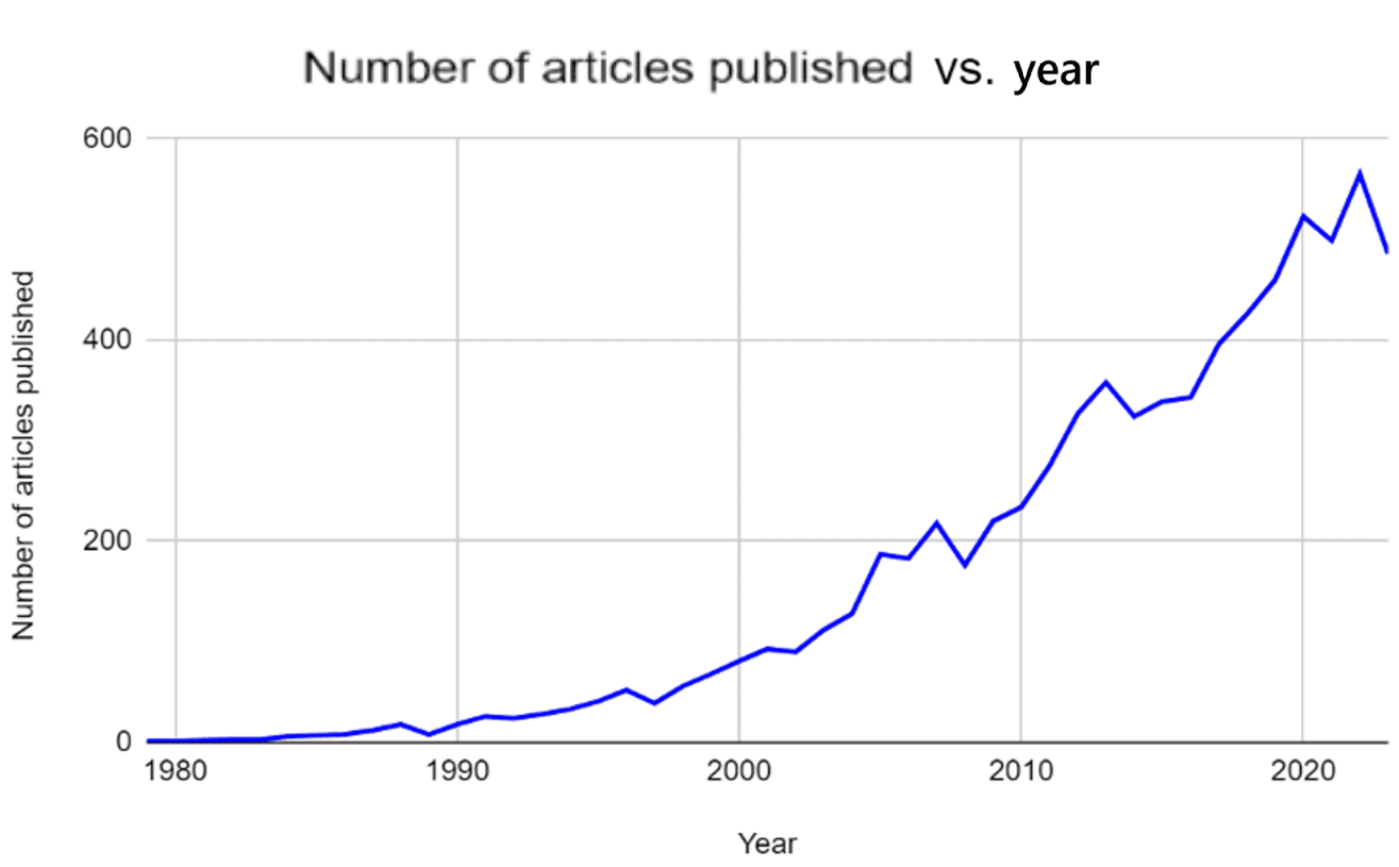
Graph displaying number of publications from 1979 to 2023.

A total of 7,710 articles were received for analysis. When examining the number of articles published about small-cell lung cancer in geriatric patients in comparison to the years recorded, it can be noted that 7,710 articles were published in total between 1979 and 2023 (Figure 1).

Analyzing this information further, only one article was published in 1979, and there was a steady increase after that. The number of articles written per year increased until it reached a maximum of 586 articles in the year of 2022, making it the greatest yield in terms of the number of papers published. After 2022, the number of papers that were published on the topic of small-cell lung cancer in the elderly decreased by about 100 articles, which equates to a 17% decrease.

**Figure 2:**
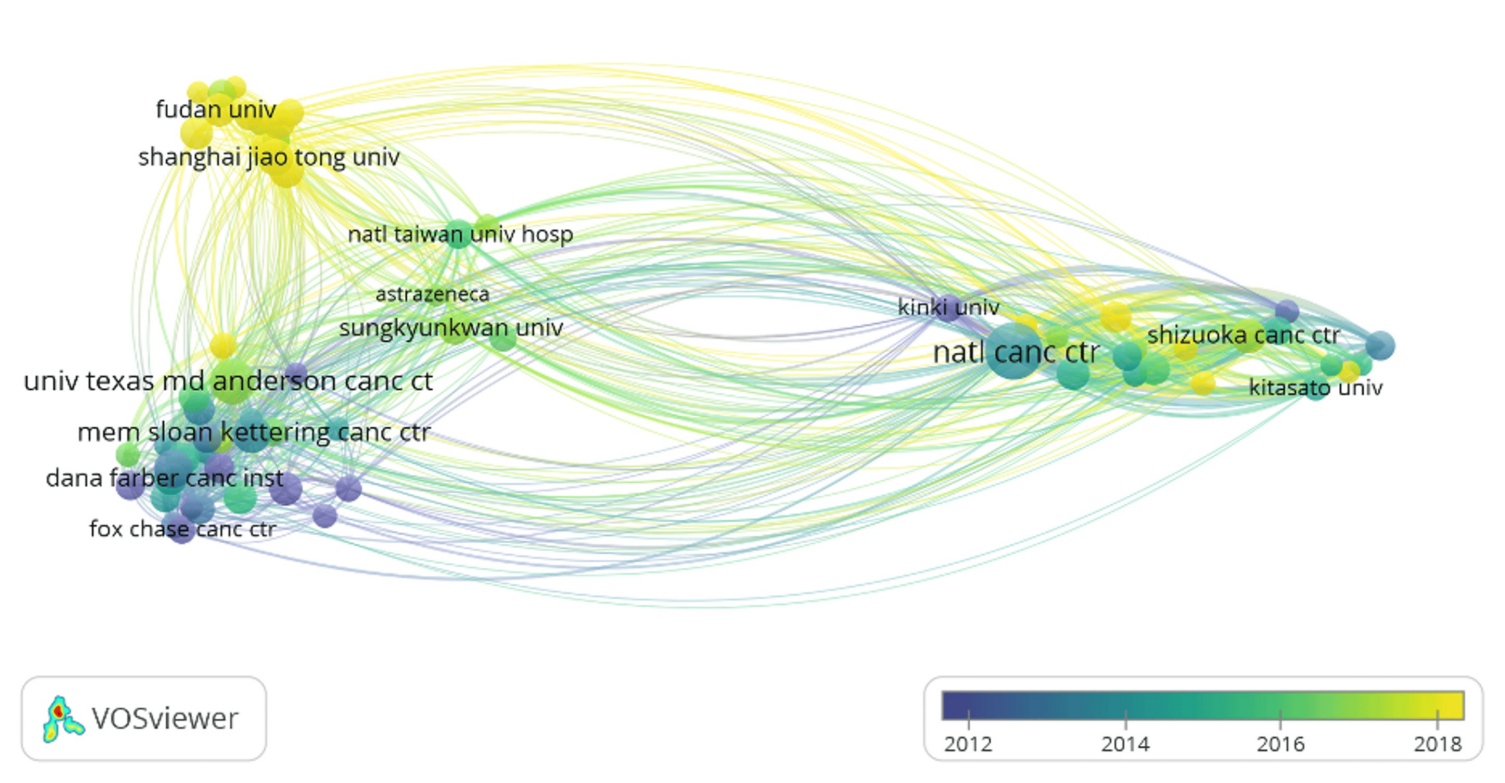
Visual of the year of publication for each of the publishing organizations. VOSviewer (Leiden, Netherlands: Leiden University)

When reviewing the different organizations that published papers about small-cell lung cancer in geriatric patients, the organization that had the most number of publications was the National Cancer Center, with a majority of its articles published around 2014-2016. Fudan University and Shanghai Jiao Tong University, located in the People’s Republic of China, have the most recent publications, but not as many as the National Cancer Center. Another organization that has many publications, a high link strength, and many co-authorships is the University of Texas MD Anderson Cancer Center. Link strength is determined by the number of times an article is cited (Figure 2).

**Figure 3:**
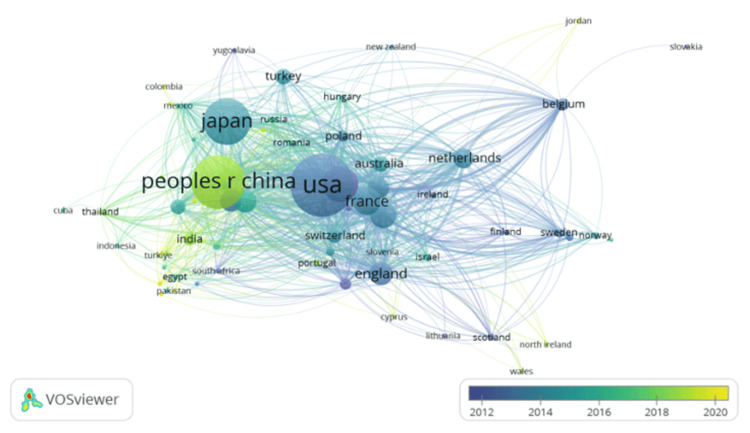
Visual of the year of publication for each of the publishing countries. VOSviewer (Leiden, Netherlands: Leiden University)

When looking at the total number of publications in terms of different countries in the field of small-cell lung cancer in geriatric patients, the 10 countries with the most publications are the United States, with 2,001 publications; the People’s Republic of China, with 1,507 publications; Japan, with 1,188 publications; Italy, with 721 publications; Germany, with 417 publications; England, with 344 publications; South Korea, with 340 publications; Canada, with 315 publications; Spain, with 313 publications; and the Netherlands, with 267 publications. The United States has the most publications, but most of its papers are older (as displayed through the blue circle labeled "USA"). The People’s Republic of China has the second most publications, and it has more recently published papers (as displayed through the bright green circle labeled "peoples r china"). Japan is third on the list of the most publications, with most of its papers published around 2016 (as displayed through the turquoise circle labeled "japan") (Figure 3).

**Figure 4:**
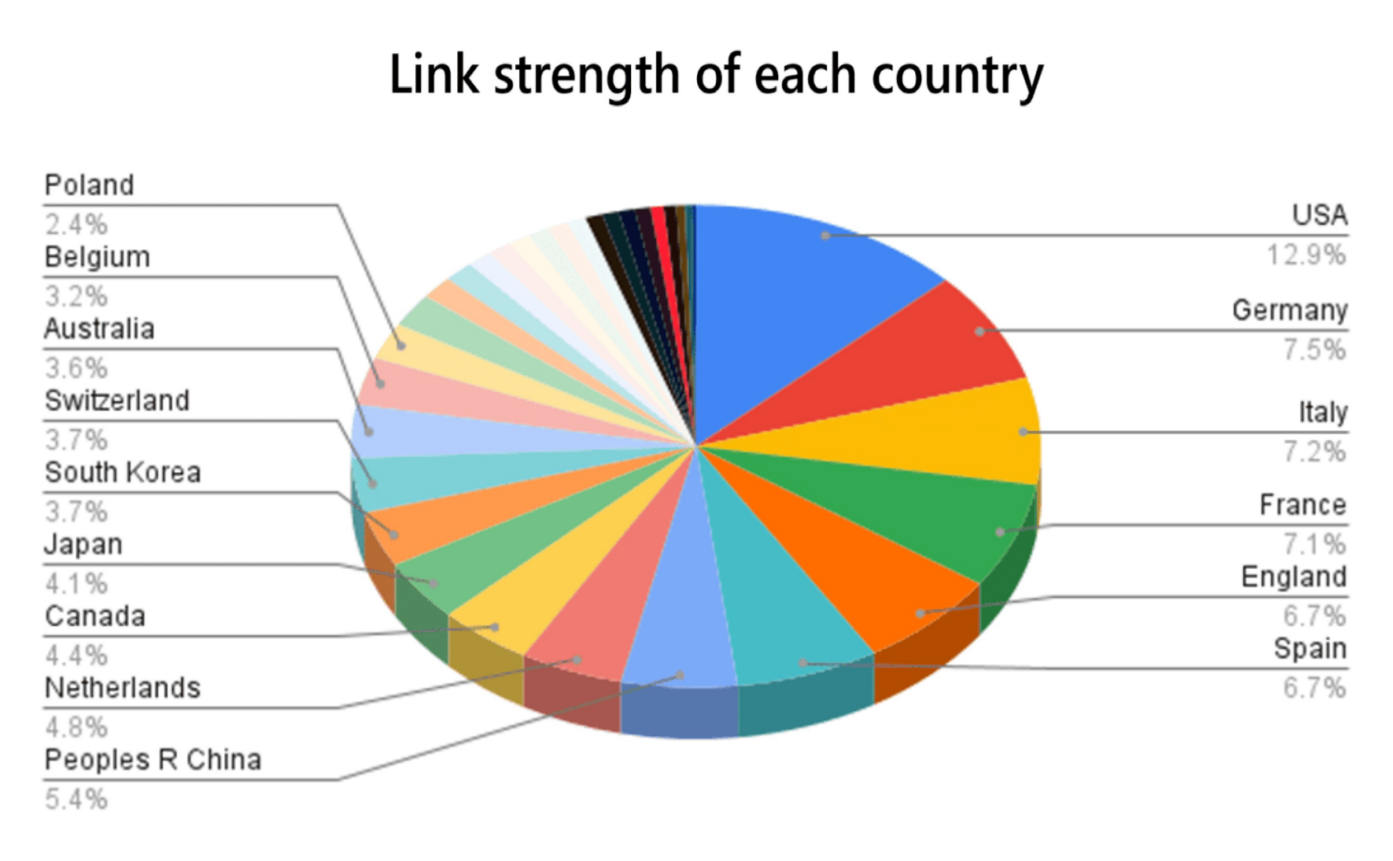
Pie graph displaying link strength of each of the publishing countries.

When looking at the link strength of different countries that published papers relating to small-cell lung cancer in geriatric patients, the three countries that had the highest link strength were the United States, Germany, and Italy. The United States had a link strength of 1,391, Germany had a link strength of 810, and Italy had a link strength of 773. Taking fourth and fifth place in terms of link strength were the People’s Republic of China with a score of 586 and Japan with a score of 443 (Figure 4). 

**Figure 5:**
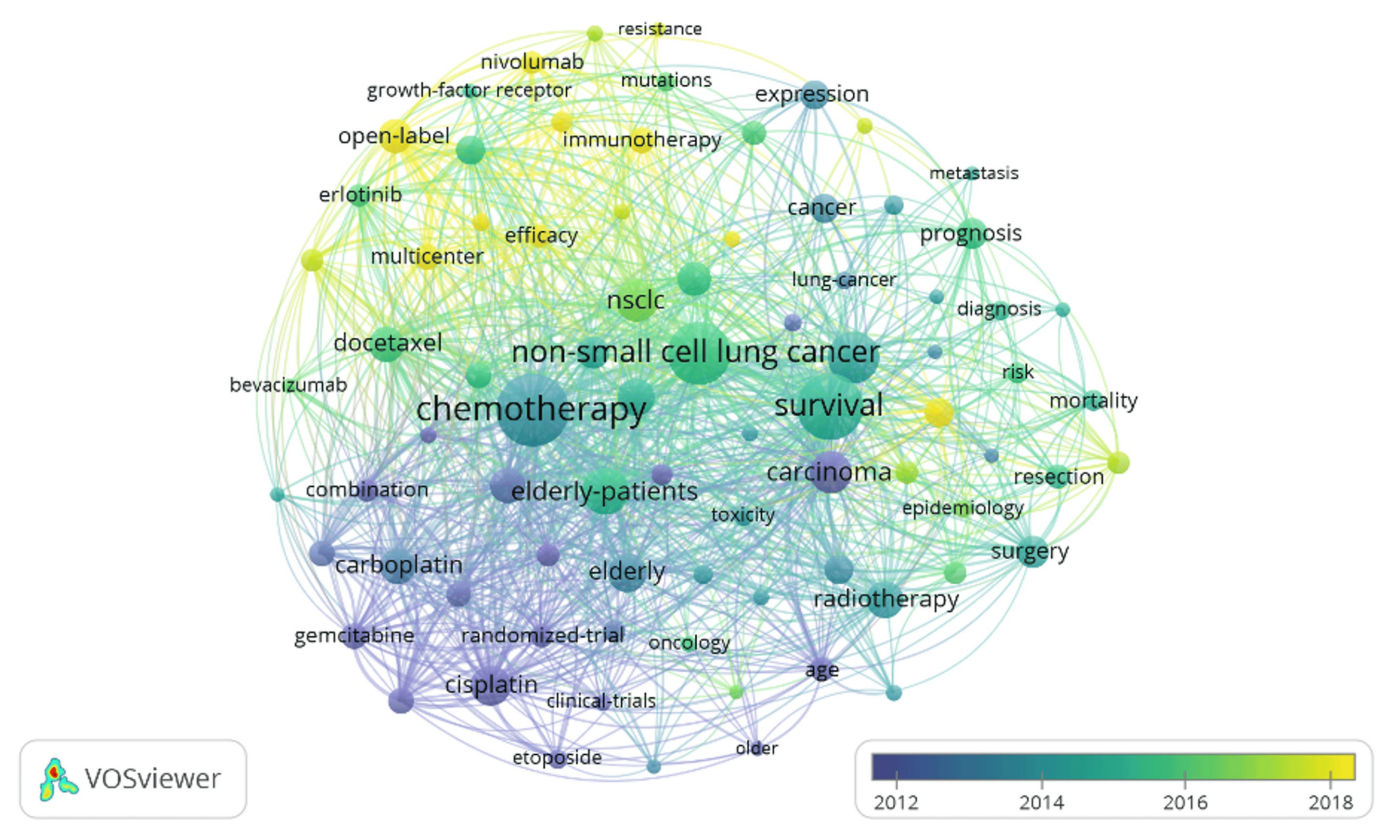
Visual of key words found in publications. VOSviewer (Leiden, Netherlands: Leiden University)

When examining the keywords associated with small-cell lung cancer in geriatric patients, the most common words are chemotherapy, survival, non-small-cell lung cancer, and lung cancer. Chemotherapy has occurred 1,744 times and has a link strength of 8,882. Survival has occurred 1,472 times and has a link strength of 6,410. Non-small-cell lung cancer occurred 1,373 times and has a link strength of 5,536. Lastly, lung cancer occurred 1,009 times and has a link strength of 3,554 (Figure 5).

Discussion

When looking at the number of articles published in regard to the year, it can be noted that there has been a steady increase in the number of publications per year since 1979. Additionally, the spike in publications in 2020 may be due to both COVID-19 as a disease and, consequently, the COVID-19 pandemic. On one hand, researchers were quarantined, which may have allowed for increased time to conduct in-depth research and publish many papers on small-cell lung cancer in geriatric populations. On the other hand, with the symptoms of COVID-19 primarily impacting the respiratory system, more research focus may have been placed on this organ system and thus other diseases that impact the respiratory system, such as small-cell lung cancer. When looking at the different organizations that published papers on small-cell lung cancer in geriatric populations, the University of Texas MD Anderson Cancer Center was the top cancer organization in the United States for research regarding small-cell lung cancer in geriatric populations. When looking at the number of publications per country, the United States has the most publications due to research centers such as the National Cancer Center, the University of Texas MD Anderson Cancer Center, and Memorial Sloan Kettering Cancer Center, all of which are located in this country. The institutions in the People’s Republic of China had the most publications per institution due to their heavy research. Some of these organizations were Shanghai Jiao Tong University and Fudan University. When looking at the link strength of all of the countries, while the US, People's Republic of China, and Japan are the top three in terms of sheer number of publications, their link strength does not parallel. The United States is the top in terms of link strength and publications, the People’s Republic of China and Japan are fourth and fifth. This may be due to the fact that these countries (People’s Republic of China and Japan) have a lot of publications, but their work is not cited very much compared to articles in the United States or Germany. The reason the United States has the highest number of publications could be because lung cancer is still the leading cause of cancer-related deaths in Western countries [11]. Also, the United States is focusing more on geriatric patients because more than two-thirds of all patients in the United States who are dying from lung cancer are over the age of 65 years [12]. Additionally, more than 50% of lung cancer patients are diagnosed when they are over the age of 65 years, and about 30% are over 70 years [13,14]. Lastly, when examining the keywords used in papers regarding small-cell lung cancer in geriatric populations, the keywords that are being used more recently are related to therapeutics. Some of these new therapeutic words are growth factor receptor, immunotherapy, and nivolumab. This shows how the topic is shifting from the disease itself to a more therapeutic approach aimed at producing new medications and treatments to remedy small-cell lung cancer.

Strengths and limitations

This study provides valuable insights into global research trends on small-cell lung cancer among older adult populations, highlighting key contributors, institutions, and emerging therapies. One of its strengths lies in the use of a robust bibliometric analysis tool, VOSviewer, and data source, Web of Science, which adds to the reliability of the findings. However, limitations include potential publication bias, as only articles indexed in the Web of Science were analyzed, possibly excluding relevant studies from other databases. Additionally, the reliance on keyword-based searches may have led to the omission of other pertinent literature not explicitly labeled with selected terms. Citation metrics such as link strength may also not fully capture the quality or impact of research outputs.

## Conclusions

This bibliometric analysis highlights a growing global research focus on small-cell lung cancer among older adults, with the United States, the People’s Republic of China, and Japan leading in publication volume. The findings show an increasing emphasis on therapeutic strategies, as reflected in the evolution of keywords used over time. Continued research efforts, particularly in the development of targeted treatments, are essential to improving outcomes for this high-risk population.
